# Prognostic Significance of baseline systemic inflammation markers in PD-L1-negative advanced non-small cell lung cancer patients treated with the BRICS sequential regimen

**DOI:** 10.3389/fimmu.2025.1686521

**Published:** 2025-12-10

**Authors:** Jianxin Chen, Ming Lin, Jian Wang, Junhui Wang, Yonghai Peng, Zongyang Yu

**Affiliations:** 1 Fuzong Clinical Medical College of Fujian Medical University, Fuzhou, Fujian, China; 2 Department of International Ward, The Quzhou Affiliated Hospital of Wenzhou Medical University, Quzhou People′s Hospital, Quzhou, Zhejiang, China; 3 Department of Oncology, Cangshan Hospital Area, 900 Hospital of the Joint Logistics Support Force, Fujian, China; 4 Department of Gastroenterology, Jiaxing Second Hospital, Jiaxing, Zhejiang, China; 5 Department of Pulmonary and Critical Care Medicine, Fuzong Clinical Medical College of Fujian Medical University & 900th Hospital of PLA Joint Logistic Support Force, Fuzhou, Fujian, China

**Keywords:** non-small cell lung cancer, C-reactive protein-to-lymphocyte ratio, lactate dehydrogenase, prognostic biomarkers, systemic inflammation markers

## Abstract

**Background:**

Non-small cell lung cancer (NSCLC) remains a leading cause of cancer mortality, with PD-L1-negative tumors exhibiting poor response to immune checkpoint inhibitors (ICIs) and chemotherapy. The multimodal BRICS regimen-integrating stereotactic body radiotherapy (SBRT), *Bifidobacterium* supplementation, low-dose chemotherapy, and PD-1 blockade—offers a promising approach for this high-risk subgroup. This study evaluates the prognostic role of baseline systemic inflammation markers in this context.

**Methods:**

A retrospective analysis included 23 PD-L1-negative (TPS <1%), EGFR/ALK wild-type advanced NSCLC patients treated with BRICS (2018–2024). Pretreatment markers (e.g., CLR, LDH) were assessed from blood samples. The sequential regimen involved: (1) SBRT (24 Gy/3 fractions); (2) oral probiotics (6 g/day); (3) nab-paclitaxel (200 mg); and (4) anti-PD-1 antibody over six 21-day cycles. Primary endpoints were progression-free survival (PFS) and overall survival (OS), analyzed via Cox regression and Kaplan-Meier curves.

**Results:**

Efficacy outcomes were robust: objective response rate 74.0%, disease control rate 95.7%, median PFS 16.0 months (95% CI: 9.11–22.89), and median OS 32.7 months (95% CI: 11.53–53.87). Univariate analysis showed elevated CLR predicted increased progression risk (HR = 2.907, p=0.04) and death risk (HR = 2.995, p=0.049), while high LDH correlated with worse PFS (HR = 3.448, p=0.013) and OS (HR = 3.016, p=0.041). Subgroup stratification confirmed shorter median PFS (7.10 vs. 20.0 months, p=0.008) and OS (9.20 vs. 36.20 months, p=0.031) for high LDH (≥250 U/L), and reduced PFS (14.20 vs. 19.10 months, p=0.032) and OS (17.70 vs. 36.20 months, p=0.038) for high CLR (≥10).

**Conclusion:**

Baseline CLR and LDH are independent prognostic biomarkers for PD-L1-negative NSCLC patients receiving BRICS, reflecting systemic inflammation that may limit efficacy. These markers could optimize patient stratification and guide personalized therapy.

## Introduction

Lung cancer remains the leading cause of cancer-related mortality globally, with non-small cell lung cancer (NSCLC) accounting for approximately 85% of cases and over 50% presenting with metastatic disease at diagnosis (1, 2). Patients lacking actionable driver mutations (e.g., EGFR, ALK) and programmed death-ligand 1 (PD-L1) expression represent a clinically challenging subgroup, exhibiting intrinsic resistance to immune checkpoint inhibitors (ICIs) and limited survival under standard platinum-based chemotherapy (3, 4). Real-world analyses confirm that nearly half of advanced NSCLC cases are PD-L1 negative (tumor proportion score <1%), a population with median overall survival (OS) rarely exceeding 12–18 months with contemporary chemoimmunotherapy (5–7). In addition to PD-L1 expression, systemic inflammation markers have emerged as potent prognostic biomarkers across NSCLC treatment paradigms, including immunotherapy and chemo-immunotherapy. The neutrophil-to-lymphocyte ratio (NLR) and platelet-to-lymphocyte ratio (PLR), derived from routine blood counts, serve as integrative indicators of the systemic inflammatory response and immune status. Elevated baseline NLR and PLR have been consistently associated with poorer survival outcomes in patients receiving ICIs (8–10). For instance, a meta-analysis confirmed that a high pretreatment NLR was predictive of reduced OS and progression-free survival (PFS) in advanced NSCLC patients treated with PD-1/PD-L1 inhibitors (8). Similarly, lactate dehydrogenase (LDH), a key enzyme in aerobic glycolysis, reflects heightened tumor metabolic activity and is often elevated in hypoxic, immunosuppressive tumor microenvironments. Elevated LDH has been established as an independent negative prognostic factor for OS in patients undergoing chemo-immunotherapy (5, 11). These markers collectively represent accessible tools for risk stratification, potentially identifying patients with heightened inflammatory or metabolic burdens that may compromise treatment efficacy, even within the challenging PD-L1-negative subgroup. This profound unmet need underscores the urgency to develop novel combinatorial strategies capable of overcoming immunologically “cold” tumor microenvironments (TMEs).

Emerging evidence suggests multimodal approaches may synergistically prime antitumor immunity. Stereotactic body radiotherapy (SBRT) induces immunogenic cell death, releasing tumor neoantigens and enhancing dendritic cell cross-presentation to T cells (12). The phase II PEMBRO-RT trial demonstrated that SBRT combined with pembrolizumab doubled objective response rates (ORR) in PD-L1-negative NSCLC compared to ICI monotherapy (13). Concurrently, gut microbiome modulation-particularly *Bifidobacterium* supplementation—augments ICI efficacy by promoting CD8+ T-cell infiltration and dendritic cell maturation (14) (15). Established evidence further indicate that low-dose metronomic chemotherapy depletes immunosuppressive regulatory T cells (Tregs) while preserving effector lymphocytes (16). These mechanisms collectively inspired the development of the ​*Bifidobacterium* supplementation, ​Radiotherapy, ​Immunotherapy (ICI), ​Chemotherapy, and ​Stereotactic approach (BRICS) sequential regimen.

In our initial publication (15), we reported promising efficacy of the BRICS regimen in 23 patients with PD-L1-negative, EGFR/ALK wild-type advanced NSCLC, achieving an ORR of 74.0%, disease control rate (DCR) of 95.7%, median PFS of 16.0 months, and median OS of 32.7 months (2). The protocol sequentially integrates: (1) SBRT (24 Gy in 3 fractions) to a single lesion to trigger antigen release; (2) high-dose probiotics (6 g/day) to sustain gut-immune axis activation; (3) low-dose nab-paclitaxel to reduce immunosuppressive cells; and (4) PD-1 blockade to amplify T-cell recognition.

Systemic inflammation markers-including NLR, C-reactive protein (CRP), and lactate dehydrogenase (LDH)-are increasingly recognized as prognostic indicators in NSCLC, reflecting tumor-associated inflammation and metabolic dysregulation. Elevated baseline inflammation correlates with impaired survival across multiple malignancies, potentially due to immunosuppressive cytokine cascades and T-cell exhaustion. However, comprehensive analyses linking pretreatment inflammatory profiles to therapeutic outcomes in PD-L1-negative NSCLC receiving multimodal immunotherapy remain limited.

Building upon our initial clinical findings (15), this study explores the prognostic significance of baseline systemic inflammation markers within the same BRICS-treated cohort. We specifically evaluate nine hematologic indices—neutrophil-to-lymphocyte ratio (NLR), platelet-to-lymphocyte ratio (PLR), lymphocyte-to-monocyte ratio (LMR), platelet-to-albumin ratio (PAR), systemic immune-inflammation index (SII), neutrophil-to-prealbumin ratio (NPR), C-reactive protein-to-albumin ratio (CAR), C-reactive protein-to-lymphocyte ratio (CLR), and C-reactive protein (CRP)—as well as serum lactate dehydrogenase (LDH), hypothesizing that these parameters may characterize survival heterogeneity despite uniform therapeutic intervention. This investigation aims to identify accessible biomarkers for patient stratification and deepen understanding of inflammation-immune interactions in NSCLC.

## Methods

### Study design and patient population​

This retrospective cohort study utilized the same patient population described in our initial publication (15), which evaluated the BRICS sequential therapeutic regimen in advanced NSCLC patients. The cohort consisted of 23 patients with PD-L1-negative (tumor proportion score <1%), EGFR/ALK wild-type advanced NSCLC, treated between January 2018 and December 2024 at Cangshan Hospital Area, 900 Hospital of the Joint Logistics Support Force, and Quzhou People’s Hospital. Inclusion criteria mirrored the original study: histologically/cytologically confirmed NSCLC, measurable lesions per RECIST v1.1, absence of actionable mutations (e.g., EGFR, ALK), and ECOG performance status ≤2. Exclusion criteria encompassed autoimmune diseases, prior immunotherapy exposure (unless part of BRICS), and incomplete biomarker data. All patients provided written informed consent, and the study was approved by the institutional review boards of Quzhou People’s Hospital and 900 Hospital, adhering to the Declaration of Helsinki.

### BRICS therapeutic protocol

**Figure 1:**
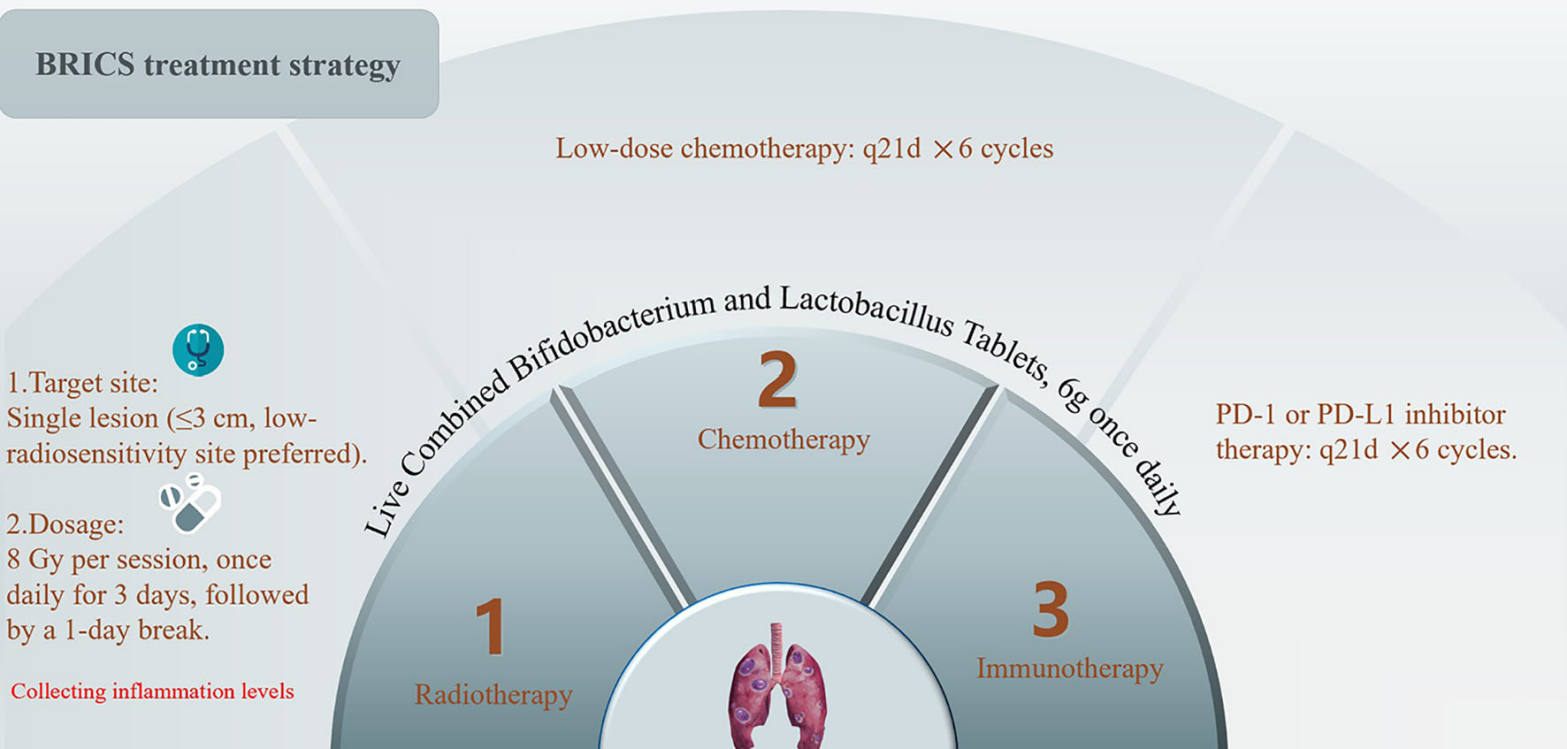
Treatment protocol of BRICS sequential therapeutic regimen.

The sequential regimen comprised four core components administered six 21-day cycles.​ SBRT​: A single metastatic lesion (≤3 cm; non-central location) received 24 Gy in 3 fractions (8 Gy/fraction daily). ​Probiotic Supplementation: Oral triple-strain *Bifidobacterium*/Lactobacillus (6 g/day) initiated concurrently with SBRT and continued indefinitely. ​Low-Dose Chemotherapy: Intravenous nab-paclitaxel (200 mg, fixed dose) administered on Day 5. ​PD-1 Inhibition: Anti-PD-1 antibody (e.g., toripalimab 240 mg) infused on Day 5. The treatment protocol is visually summarized in **Figure 1**.

### ​Assessment of systemic inflammation markers​

Baseline systemic inflammation markers were evaluated prior to BRICS regimen initiation using peripheral blood samples collected within one week before treatment commencement. The markers included: Neutrophil-to-Lymphocyte Ratio (NLR), Calculated as absolute neutrophil count divided by absolute lymphocyte count. ​Platelet-to-Lymphocyte Ratio (PLR)​: Derived from platelet count divided by lymphocyte count. ​Lymphocyte-to-Monocyte Ratio (LMR)​: Lymphocyte count divided by monocyte count. ​Platelet-to-Albumin Ratio (PAR)​: Platelet count divided by serum albumin level. ​Systemic Immune-Inflammation Index (SII)​: Computed as (platelet count × neutrophil count)/lymphocyte count. ​Neutrophil-to-Platelet Ratio (NPR)​: Neutrophil count divided by platelet count. ​C-reactive Protein-to-Albumin Ratio (CAR)​: Serum CRP level divided by albumin level. ​C-reactive Protein-to-Lymphocyte Ratio (CLR)​: CRP level divided by lymphocyte count. ​C-reactive Protein (CRP)​: Measured via immunoturbidimetry. ​Lactate Dehydrogenase (LDH)​: Assessed using enzymatic methods.

### ​Outcome measures​

Primary efficacy outcomes were PFS and OS, defined as per the initial study: PFS as the time from BRICS initiation to disease progression or death, and OS as the time to death from any cause. Secondary outcomes included ORR and DCR, evaluated via CT/MRI scans every 8 weeks using RECIST v1.1. Survival data were censored at the last follow-up (December 31, 2024).

### Statistical analysis for inflammation marker correlations​

Correlations between baseline inflammation markers and survival outcomes were analyzed using a multi-tiered statistical approach: ​Univariate Analysis: Cox proportional hazards regression models assessed the impact of each inflammation marker on PFS and OS. Hazard ratios (HRs) with 95% confidence intervals (CIs) were computed, and Kaplan-Meier survival curves were generated for visualization. Log-rank tests determined statistical significance (p < 0.05). Subgroup Analysis: Patients were stratified by CLR and LDH levels. Log-rank tests compared survival curves between subgroups, and interaction p-values were reported to evaluate heterogeneity. Given the cohort size (n=23) and limited event counts (19 progression events, 14 deaths), multivariable adjustment was intentionally excluded. The event-per-variable ratio (≤2.1 events/marker) falls below the minimum threshold (EPV≥10) required for reliable Cox modeling, which would fundamentally constrain statistical validity and risk overfitted hazard ratios. All analyses were performed using SPSS v23.0 (SPSS Inc., Chicago, USA), with two-sided p-values <0.05 considered significant.

## Results

### Patient characteristics and outcomes

A total of 23 patients were included in this study. The characteristics of the patients are summarized in **Table 1**. The median age was 62.0 years. Most patients were male (82.6%), no history of smoking (69.6%), and were classified as TNM stage IV (78.3%). The number of metastatic organs was as follows: 0 (21.7%), 1 (34.8%), and ≥2 (43.5%). Additionally, 91.3% of patients had a PS score of <2. Peripheral blood analysis prior to BRICS regimen initiation revealed characteristic profiles of systemic inflammation markers across the cohort of 23 PD-L1-negative NSCLC patients. Key ratios included a mean NLR of 3.47 ± 0.33, Platelet-to-Lymphocyte Ratio (PLR) of 188.86 ± 16.84, and C-reactive Protein-to-Lymphocyte Ratio (CLR) of 15.82 ± 5.23. Lactate Dehydrogenase (LDH) levels averaged 277.57 ± 39.10 U/L, reflecting baseline metabolic activity within the cohort.

**Table 1:** Baseline characteristics.

Baseline characteristics	All patients (n = 23)
Age (years), n (%)	
Median (range)	62 (54-67)
≥60	15 (65.2)
<60	8 (34.8)
Gender, n (%)	
Male	19 (82.6)
Female	4 (17.4)
TNM stage, n (%)	
III	5 (21.7)
IV	18 (78.3)
Smoking status, n (%)	
Nonsmoker	16 (69.6)
Former smoker/smoker	7 (30.4)
Number of metastatic organs, n (%)	
0	5 (21.7)
1	8 (34.8)
≥ 2	10 (43.5)
ECOG PS, n (%)	
0–1	21 (91.3)
2	2 (8.7)
Level of systemic inflammation, (mean ± *SD*)	
NLR	3.47 ± 0.33
PLR	188.86 ± 16.84
LMR	3.33 ± 0.30
PAR	5.83 ± 0.53
SII	791.64 ± 89.84
NPR	0.34 ± 0.32
CAR	0.43 ± 0.11
CLR	15.82 ± 5.23
CRP (mg/L)	16.72 ± 4.30
LDH (U/L)	277.57 ± 39.10

NLR, Neutrophil-to-Lymphocyte Ratio; PLR, Platelet-to-Lymphocyte Ratio; LMR, Lymphocyte-to-Monocyte Ratio; PAR, Platelet-to-Albumin Ratio; SII, Systemic Immune-Inflammation Index (Platelets × Neutrophils/Lymphocytes); NPR, Neutrophil-to-Platelet Ratio; CAR, C-reactive Protein-to-Albumin Ratio; CLR, C-reactive Protein-to-Lymphocyte Ratio; CRP, C-reactive Protein; LDH, Lactate Dehydrogenase; ECOG PS, Eastern Cooperative Oncology Group performance status.

### Efficacy outcomes consistent with primary study​

Efficacy endpoints aligned with our initial BRICS regimen publication (15): ORR was 74.0% (17/23), DCR 95.7%, median PFS 16.0 months (95% CI: 9.11–22.89), and median OS 32.7 months (95% CI: 11.53–53.87). All the results of efficacy were represented in the Appendix1.

### Univariate survival analysis of inflammation markers​

**Figure 2:**
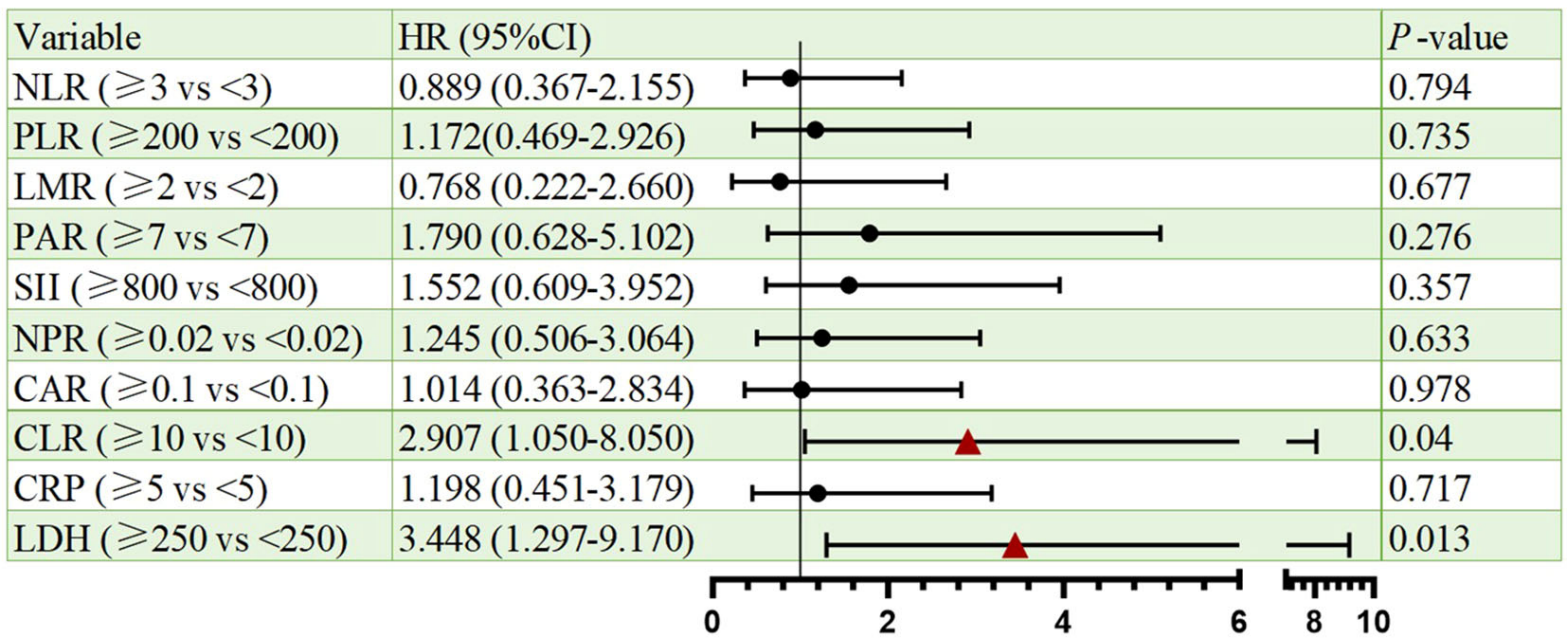
Forest plots of univariate Cox regression analyses for PFS. Hazard ratios (HRs) and 95% confidence intervals are shown. Statistical significance was defined as p < 0.05.

**Figure 3:**
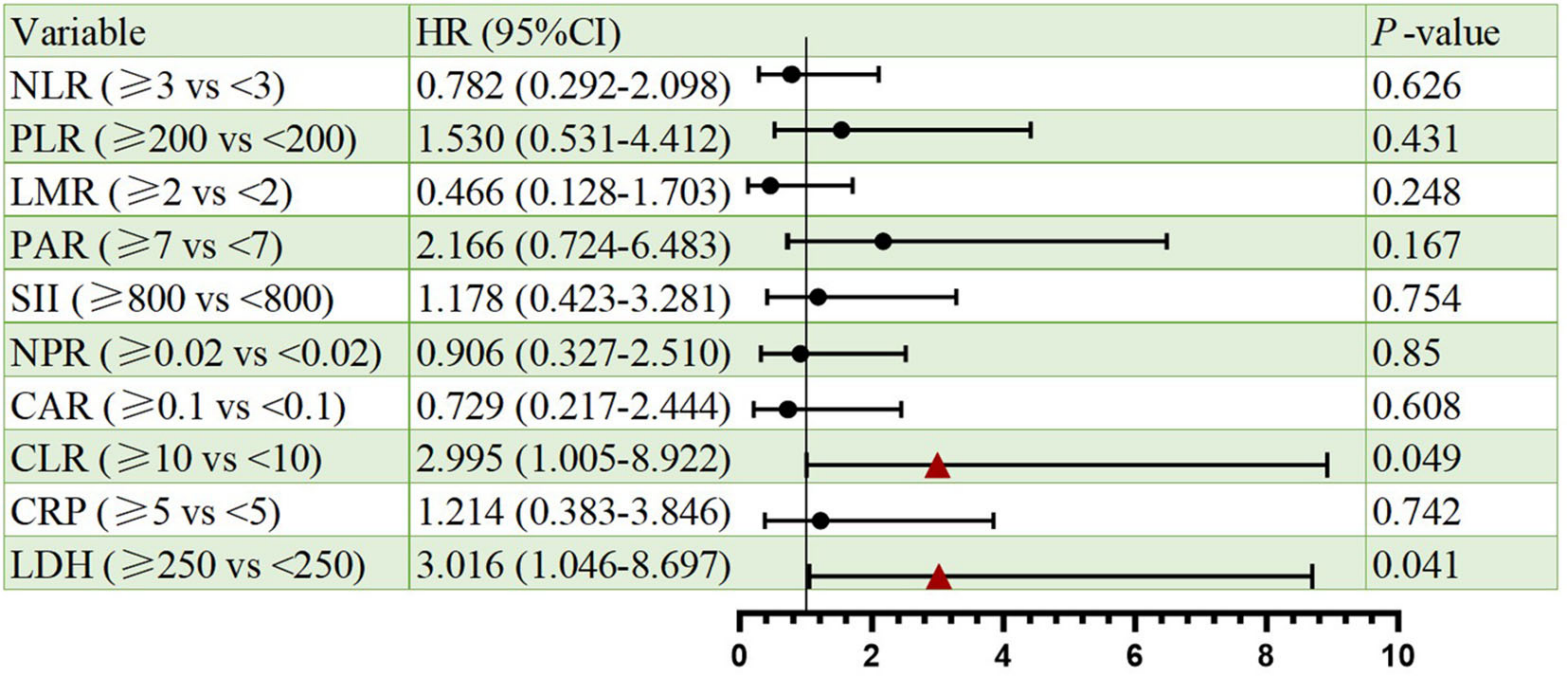
Forest plots of univariate Cox regression analyses for OS. Hazard ratios (HRs) and 95% confidence intervals are shown. Statistical significance was defined as p < 0.05.

Cox proportional hazards regression identified CLR and LDH as significant prognostic indicators for both PFS and OS. Elevated CLR was associated with a significantly increased risk of progression per unit rise (HR = 2.907, 95% CI: 1.050–8.050, p=0.04), as well as increased risk of death (HR = 2.995 CI: 1.005-8.922, p=0.049). Similarly, high LDH predicted poorer PFS (HR = 3.448, 95% CI: 1.297–9.170, p=0.013) and OS (HR = 3.016, 95% CI: 1.046–8.697, p=0.041). Besides, other markers (NLR, PLR, LMR, PAR, SII, NPR, CAR, CRP) lacked statistical significance. All the results were shown in **Figures 2**, **3**.

### Subgroup survival by LDH and CLR stratification

**Figure 4:**
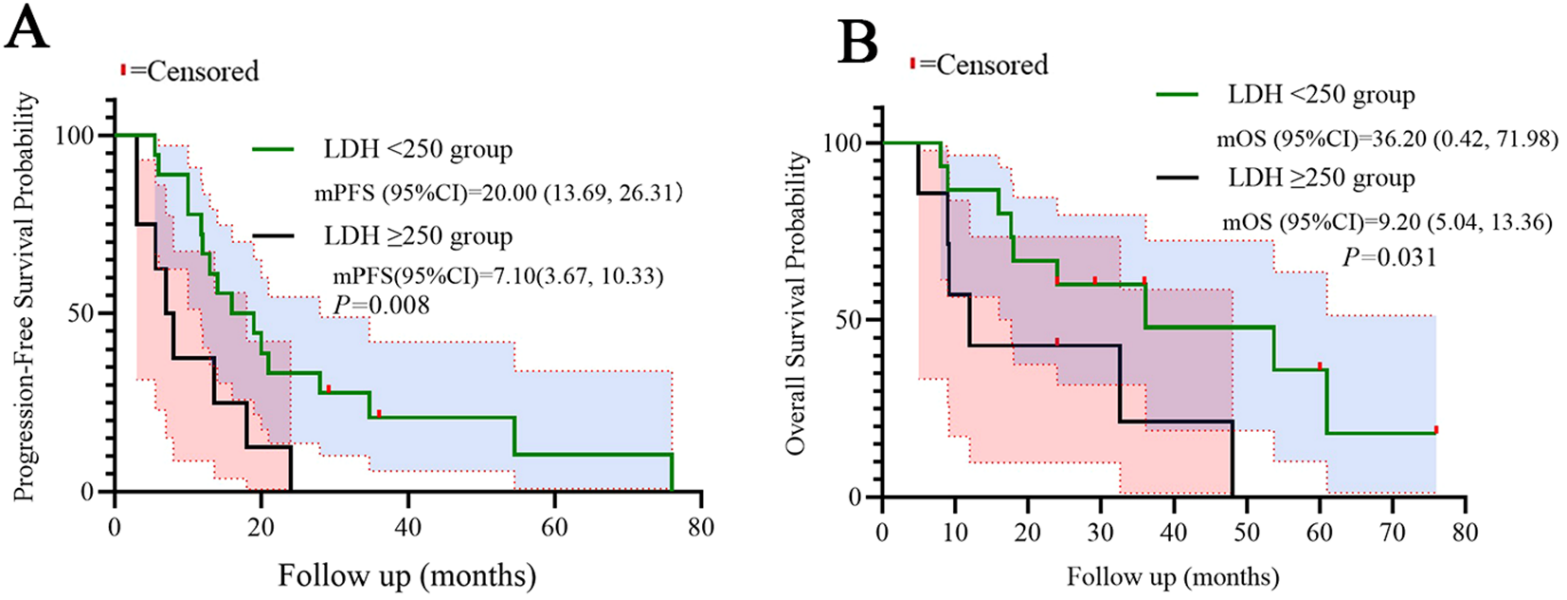
Kaplan-Meier curves for PFS **(A)** and OS **(B)** stratified by LDH. Statistical significance was defined as p < 0.05.

**Figure 5:**
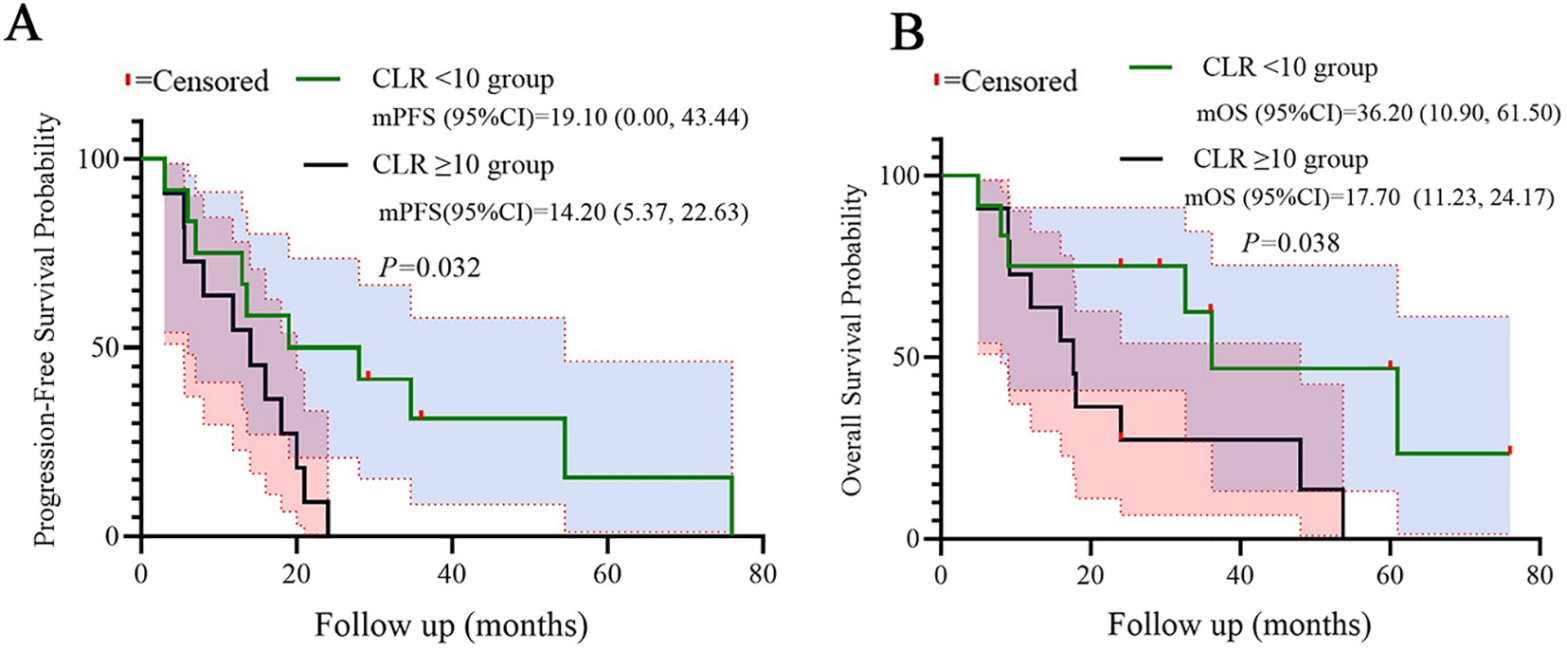
Kaplan-Meier curves for PFS **(A)** and OS **(B)** stratified by CLR. Statistical significance was defined as p < 0.05.

Patients with high LDH (≥250 U/L) exhibited significantly shorter median PFS (7.10 vs. 20.0 months, log-rank p=0.008) and OS (9.20 vs. 36.20 months, log-rank p=0.031) compared to low-LDH counterparts. Similarly, high CLR (≥10) correlated with reduced PFS (14.20 vs. 19.10 months, p=0.032) and OS (17.70 vs. 36.20 months, p=0.038). Kaplan-Meier curves demonstrated distinct separation between subgroups, which were shown in the **Figures 4**, **5**.

## Discussion

Our study provides evidence that baseline systemic inflammation markers, particularly C-reactive protein-to-lymphocyte ratio (CLR) and lactate dehydrogenase (LDH), serve as robust prognostic biomarkers in PD-L1-negative advanced NSCLC patients receiving the multimodal BRICS sequential regimen. The key findings demonstrate that elevated CLR (≥10) and LDH (≥250 U/L) correlate with significantly reduced PFS (median PFS: 14.2 vs. 19.1 months for high vs. low CLR; 7.1 vs. 20.0 months for high vs. low LDH) and overall survival (median OS: 17.7 vs. 36.2 months and 9.2 vs. 36.2 months, respectively). These results extend our initial report on BRICS efficacy by identifying accessible biomarkers that stratify survival outcomes in this immunotherapy-resistant population (15).

The survival outcomes observed in our BRICS-treated cohort-median PFS of 16.0 months and median OS of 32.7 months-compare favorably with historical data for PD-L1-negative advanced NSCLC. For context, the standard first-line therapy for this population, platinum-based chemoimmunotherapy as exemplified by the KEYNOTE-189 regimen (pembrolizumab plus pemetrexed and platinum), yields a median OS of approximately 12–18 months in similar patient groups (5, 6). This direct comparison suggests a potential survival advantage offered by the multimodal BRICS sequential strategy. The potential efficacy is likely attributable to the synergistic mechanisms engineered within the BRICS regimen, which are specifically designed to convert immunologically ‘cold’ PD-L1-negative tumors into ‘hot’ or more responsive microenvironments. Firstly, the initial application of SBRT induces immunogenic cell death, releasing a burst of tumor neoantigens that primes a *de novo* anti-tumor immune response, an effect supported by the PEMBRO-RT trial which showed enhanced response rates with radiotherapy-ICI combination (13). Secondly, concurrent high-dose probiotic supplementation, particularly *Bifidobacterium*, is posited to modulate the gut-immune axis, potentially enhancing dendritic cell function and CD8+ T-cell infiltration, as suggested by foundational microbiome studies (14, 15). Thirdly, the strategic use of low-dose nab-paclitaxel on Day 5 is intended to selectively deplete immunosuppressive regulatory T cells (Tregs) (17, 18), thereby reducing a major barrier to effective immune effector function. The sequential nature of BRICS-activating immunity (SBRT/probiotics), reducing suppression (chemotherapy), and then unleashing T-cells (anti-PD-1)-may create a more favorable and sustained immunologic sequence compared to concurrent chemoimmunotherapy, where chemotherapy’s immunosuppressive effects might concurrently dampen nascent immune responses. This multi-pronged attack on the tumor microenvironment may explain the pronounced improvement in survival outcomes observed in our study, positioning BRICS as a promising investigational approach for this high-risk population.

The prognostic value of CLR aligns with emerging evidence linking systemic inflammation to impaired antitumor immunity. Elevated CLR integrates two critical biological processes: C-reactive protein (CRP) elevation reflects IL-6-driven hepatic synthesis in response to tumor-associated inflammation (19), while lymphocytopenia indicates immunosuppression and reduced cytotoxic T-cell activity (20). Our data corroborate findings from reported researches., which demonstrated that CRP/lymphocyte composites predicted poor outcomes in cancer patients receiving ICIs treatment (21–24). The mechanistic basis lies in CRP’s role in promoting myeloid-derived suppressor cell (MDSC) expansion and PD-L1 upregulation, while lymphocytopenia limits CD8+ T-cell-mediated tumor control (25).

LDH elevation, observed in 56.5% of our cohort, signifies enhanced aerobic glycolysis (Warburg effect) and tumor hypoxia-conditions favoring immunosuppressive TME remodeling (24, 26, 27). High LDH correlates with increased adenosine production, which inhibits dendritic cell maturation and fosters Treg infiltration (28). Meta-analysis of confirmed that baseline LDH >ULN independently predicted worse OS across treatment modalities (8, 11). Our results specifically extend this paradigm to multimodal immunotherapy, suggesting that metabolic dysregulation may limit BRICS efficacy despite its immunomodulatory design.

Our findings align with and extend the growing body of evidence underscoring the prognostic value of systemic inflammation markers in NSCLC patients treated with modern therapies. The prognostic significance of CLR and LDH in our cohort receiving the multimodal BRICS regimen resonates with established literature on simpler ratios. For example, the prognostic weight of CLR is conceptually consistent with the well-documented roles of its components: CRP, an acute-phase reactant driven by pro-inflammatory cytokines like IL-6, and lymphocytes, essential for adaptive anti-tumor immunity. Studies by Sheng et al. and Fu et al. have demonstrated the prognostic utility of CLR and similar CRP-based ratios in lung cancer patients receiving immunotherapy or chemotherapy, linking their elevation to an immunosuppressive state (21, 23). Similarly, the strong prognostic performance of LDH in our study is highly consistent with prior meta-analyses. Publications have confirmed that elevated LDH is a robust predictor of poor outcomes in NSCLC patients treated with both ICIs and chemo-immunotherapy, likely due to its association with the Warburg effect, tumor hypoxia, and an immunosuppressive microenvironment rich in adenosine and MDSC (26). However, our study provides a novel insight by demonstrating that these markers retain their powerful prognostic capacity even within the intensive, multimodal BRICS regimen, indicating that the negative impact of baseline systemic inflammation and metabolic dysregulation is profound and may not be fully overcome by radiotherapy and microbiome modulation alone. This highlights the need for integrated strategies targeting these underlying biological processes.

While inflammation markers have been studied in conventional chemoimmunotherapy (9, 10), their role in radiotherapy-microbiome-chemotherapy combinations remains underexplored. In the present study, our CLR/LDH findings suggests that in BRICS-treated patients, systemic inflammation may override microbiome-mediated immunostimulation. Mechanistically, three factors may explain this observation. ​(1) Radiotherapy-induced inflammation: SBRT triggers acute-phase responses that transiently elevate CRP and neutrophils, potentially counteracting *Bifidobacterium*’s immunoenhancing effects. (2) ​LDH-lactate axis: Tumor-derived lactate inhibits histone deacetylases, suppressing IFN-γ production in CD8+ T cells (29). High-baseline LDH may thus create a lactic acid-rich TME resistant to PD-1 blockade. (3) ​Chemotherapy timing: Metronomic nab-paclitaxel depletes Tregs (17, 18, 30), but its Day 5 administration may occur too late to mitigate pretreatment inflammation.

The stratification power of CLR/LDH highlights a potential “inflammatory phenotype” in PD-L1-negative NSCLC, characterized by: ​Constitutive NF-κB activation: Driving IL-6/STAT3 signaling and CRP production (31, 32). ​Hypoxia-inducible factor (HIF) stabilization: Enhancing LDH-A expression and glycolytic flux (33), and ​lymphocyte exhaustion: Reduced proliferative capacity despite PD-1 inhibition (34). Our data suggest similar mechanisms may operate even when combining ICIs with radiotherapy and probiotics.

Several limitations warrant acknowledgment in the present study. ​Firstly, sample size constraints​ (n=23) limit multivariate adjustment for confounding variables. ​Retrospective design​ introduces selection bias, though consistent inclusion criteria with our primary study mitigate this concern. ​Secondly, lack of serial biomarker measurements​ prevents assessment of dynamic changes during BRICS therapy. ​Absence of cytokine profiling​ (e.g., IL-6, TGF-β) hinders mechanistic interpretation of inflammation drivers. Finally, heterogeneous SBRT targets​ (primary vs. metastatic lesions) may influence abscopal effects.

Beyond their prognostic validation, our findings on CLR and LDH carry immediate and practical implications for personalizing therapy in PD-L1-negative advanced NSCLC. We propose a structured clinical decision-pathway to integrate these readily accessible biomarkers into routine practice for patients being considered for the BRICS regimen or similar multimodal immunotherapies. First, at baseline assessment, CLR (using a cut-off of ≥10) and LDH (using a cut-off of ≥250 U/L) should be calculated from routine peripheral blood tests to stratify patients into distinct risk categories. Patients with low levels of both markers (low CLR/low LDH) represent an optimal subgroup who are most likely to derive significant long-term benefit from the full BRICS sequential regimen, as evidenced by their superior median PFS of 19.1 months and OS of 36.2 months. Conversely, patients exhibiting high levels of either or both markers (high CLR and/or high LDH) should be identified as having a high-risk “inflammatory phenotype” with a potentially suppressed immune microenvironment. For these patients, whose median OS was notably shorter (9.2-17.7 months), the standard BRICS approach may be insufficient. This identification should trigger a treatment adaptation strategy. Potential approaches include: (1) intensification of the BRICS regimen itself, for instance, by adding targeted anti-inflammatory agents (e.g., IL-6 inhibitors) to mitigate the protumor inflammatory drive before or during treatment; or (2) closer monitoring and early switch to alternative therapeutic strategies upon signs of progression. Furthermore, serial monitoring of these markers during therapy could serve as a dynamic pharmacodynamic indicator, where a failure of CLR/LDH to decrease might signal primary resistance and necessitate earlier intervention. The stratification power of CLR and LDH, as clearly visualized in our Kaplan-Meier curves (**Figures 4** and **5**), provides a straightforward, cost-effective tool for oncologists to optimize patient selection and tailor therapeutic intensity. Future prospective trials should formally validate this proposed decision-pathway and explore combinatorial strategies specifically designed to overcome the adverse prognosis associated with high baseline systemic inflammation.

In summary, our study establishes CLR and LDH as clinically accessible biomarkers that predict survival heterogeneity in PD-L1-negative NSCLC patients receiving the BRICS sequential regimen. The inverse correlation between baseline inflammation and outcomes underscores the need to address systemic immunosuppression even in multimodal therapy contexts. Future directions should include: Prospective validation in larger cohorts. Exploration of inflammation-targeting strategies. Investigation of sequential biomarker dynamics to guide adaptive therapy. The integration of inflammation markers into patient selection frameworks could optimize BRICS deployment, personalizing multimodal immunotherapy for NSCLC’s most challenging subgroup.

## Data Availability

The original contributions presented in the study are included in the article/**Supplementary Material**, Further inquiries can be directed to the corresponding author/s.
