# Supplementary material for: Prognostic Significance of baseline systemic inflammation markers in PD-L1-negative advanced non-small cell lung cancer patients treated with the BRICS sequential regimen
Source: Front Immunol. 2025 Dec 10;16:1686521. doi: 10.3389/fimmu.2025.1686521 (PMC12728061; doi:10.3389/fimmu.2025.1686521)
Supplement: Supplementary file 1 [file DataSheet1.docx]

**Table 2. Efficacy of BRICS regimen in advanced NSCLC patients (n = 23).**

| **Efficacy** | **All patients (n = 23)** |
| --- | --- |
| Complete response (%) | 0 |
| Partial response (%) | 17 (74.0) |
| Stable disease (%) | 5 (21.7) |
| Progressive disease (%) | 1 (4.3) |
| Objective response rate (%, CR, PR) | 17 (74.0) |
| Disease control rate (%, CR, PR, SD) | 22 (95.7) |
| median progression-free survival (months, 95% CI) | 16.00 (9.11, 22.89) |
| median Overall Survival (months, 95% CI) | 32.70 (11.53, 53.87) |


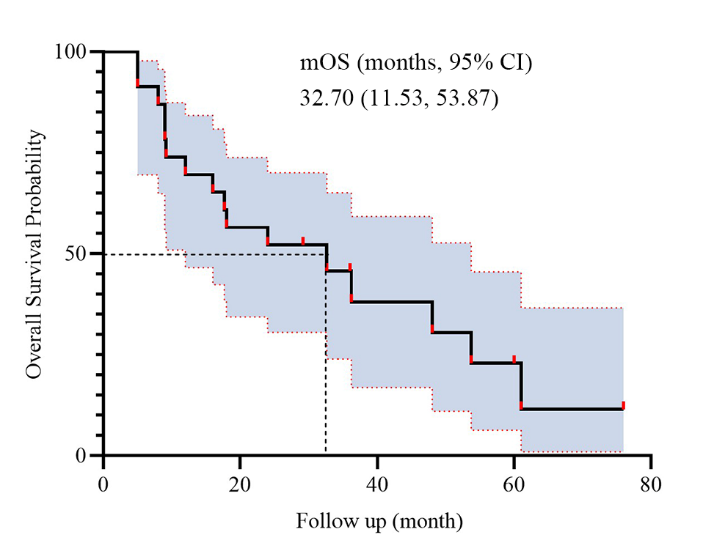


Figure 1. Kaplan-Meier survival curves of PFS in 23 patients.


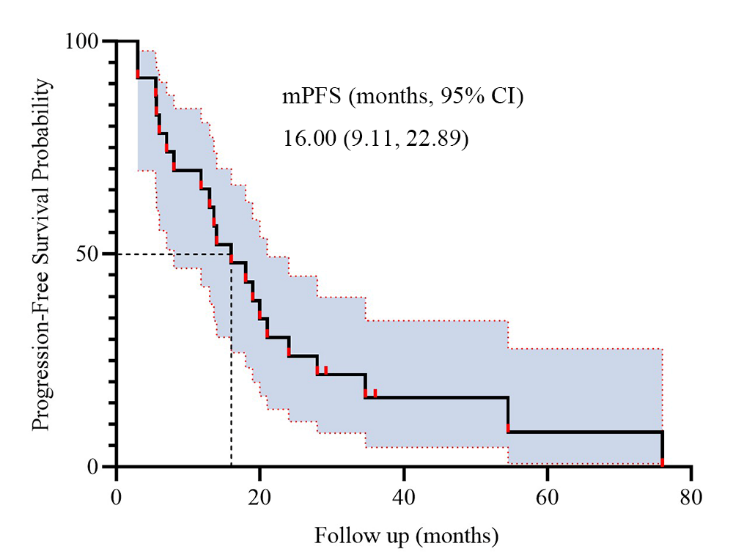


Figure 3. Kaplan-Meier survival curves of OS in 23 patients.
